# Advances in the Development of PET Ligands Targeting Histone Deacetylases for the Assessment of Neurodegenerative Diseases

**DOI:** 10.3390/molecules23020300

**Published:** 2018-01-31

**Authors:** Tetsuro Tago, Jun Toyohara

**Affiliations:** Research Team for Neuroimaging, Tokyo Metropolitan Institute of Gerontology, 35-2 Sakae-cho, Itabashi-ku, Tokyo 173-0015, Japan; tago@pet.tmig.or.jp

**Keywords:** positron emission tomography, histone deacetylase, radioligand, imaging, neurodegenerative disease

## Abstract

Epigenetic alterations of gene expression have emerged as a key factor in several neurodegenerative diseases. In particular, inhibitors targeting histone deacetylases (HDACs), which are enzymes responsible for deacetylation of histones and other proteins, show therapeutic effects in animal neurodegenerative disease models. However, the details of the interaction between changes in HDAC levels in the brain and disease progression remain unknown. In this review, we focus on recent advances in development of radioligands for HDAC imaging in the brain with positron emission tomography (PET). We summarize the results of radiosynthesis and biological evaluation of the HDAC ligands to identify their successful results and challenges. Since 2006, several small molecules that are radiolabeled with a radioisotope such as carbon-11 or fluorine-18 have been developed and evaluated using various assays including in vitro HDAC binding assays and PET imaging in rodents and non-human primates. Although most compounds do not readily cross the blood-brain barrier, adamantane-conjugated radioligands tend to show good brain uptake. Until now, only one HDAC radioligand has been tested clinically in a brain PET study. Further PET imaging studies to clarify age-related and disease-related changes in HDACs in disease models and humans will increase our understanding of the roles of HDACs in neurodegenerative diseases.

## 1. Introduction

Epigenetics is defined as mitotically and/or meiotically heritable changes in gene expression without alternations in the DNA sequence [1]. Several studies are actively examining the mechanisms of epigenetic regulation, including DNA methylation, histone modification, and RNA-based mechanisms [2]. In mammals, DNA methylation mainly involves the covalent addition of a methyl group at the 5-position of a cytosine followed by a guanine (5′-CpG-3′) [3]. This modification results in gene silencing by direct and/or indirect inhibition of transcription factor-DNA interactions. Mechanisms of DNA methylation are divided into two classes according to the corresponding DNA methyltransferase (DNMT): maintenance methylation carried out by DNMT1 and de novo methylation carried out by DNMT3a and DNMT3b [4,5]. Histone posttranslational modifications include acetylation, methylation, ubiquitination, and phosphorylation [6]. Acetylation of histones is one of the most highly studied processes and is regulated by opposing enzymes: histone acetyl transferases and histone deacetylases (HDACs) [7]. Acetylation of lysine residues by histone acetyl transferases neutralizes the positive charge of histones and consequently decreases the interaction of histones with the negatively charged phosphate group of DNA. Then, the chromatin structure becomes relaxed, allowing easier access of transcription factors to DNA. Conversely, deacetylation of histones by HDACs induces gene silencing [8,9]. Histone methylation occurs mainly on lysine and arginine residues. These residues can be methylated multiple times (lysine: three times; arginine: twice), making the effects on gene regulation complex [1,7]. Diverse classes of RNA also regulate gene expression [10]. For example, small interfering RNAs directed to promoter regions result in transcriptional gene silencing through heterochromatin formation [11].

These epigenetic modifications have emerged as key factors in functions of the central nervous system (CNS) and in the development of common neurodegenerative diseases. So far, several studies into the role of epigenetics in CNS functions (e.g., learning and memory processes, fear memory formation, and drug addiction) have been reported [8,12,13,14,15]. Meanwhile, neurodegenerative diseases that may involve epigenetic alterations include Alzheimer’s disease (AD), Parkinson’s disease, and Huntington’s disease [2]. For example, significantly reduced levels of DNA methylation were observed in temporal neocortex neuronal nuclei in an AD monozygotic twin compared to his normal sibling [16]. Understanding the expression of enzymes associated with epigenetics and associated alterations in the human brain will help elucidate the pathological mechanisms of these neurodegenerative diseases and will accelerate development of therapeutic agents targeting epigenetics.

The noninvasive imaging technique, positron emission tomography (PET), has received increasing attention in the past few decades, and now PET can be used to monitor various pathological changes in the living brain with a neurodegenerative disease [17,18]. This review summarizes the recent advances in development of PET ligands for imaging HDAC, as a representative example of an epigenetic enzyme, in the brain. With the aim of radiolabeling compounds with affinity for HDACs, various labeling methods using radioisotopes such as carbon-11 and fluorine-18 were investigated in previous studies. Unfortunately, even though more than 20 HDAC imaging radioligands have been developed since 2006, only one report describing imaging results in humans has been published. Summarizing the current advances and issues with HDAC PET ligands will help with future development of imaging techniques and will enable monitoring of the HDAC state in neurodegenerative diseases.

## 2. HDACs in the Brain

In humans, 18 HDACs can be classified into five classes based on their characteristics and homology to yeast HDACs: class I (HDAC1, 2, 3, and 8), class IIa (HDAC4, 5, 7, and 9), class IIb (HDAC6 and 10), class III (sirtuins, SIRT1–7), and class IV (HDAC11) [9,19]. Generally, class III sirtuins, which are nicotinamide adenine dinucleotide-dependent deacetylases, are considered as separate from other HDAC classes, which have zinc-dependent catalytic activity. Class I HDACs mainly reside in the nucleus and are ubiquitously expressed. Class II HDACs shuttle between the nucleus and the cytoplasm and deacetylate non-histone proteins [20]. Compared to class I HDACs, class II HDACs show tissue specificity, and consequently, class II HDAC knockout mice tend to show local defects [21]. HDAC11, the only class IV HDAC, contains conserved residues in the catalytic core regions shared by both class I and II HDACs [22].

The distribution and expression of HDACs in normal and neurodegenerative brains of mammals, including humans, have been reported. Broide et al. conducted comprehensive gene expression mapping of the 11 HDAC isoforms in the rat brain using high-resolution in situ hybridization in 2007 [23]. The distribution of HDAC subtypes showed overlapping and distinct patterns, suggesting that HDACs play distinct physiological roles in the brain. Of the 11 isoforms, HDAC3, 4, 5, and 11 demonstrated the highest expression in the brain, particularly in the cortex. In addition, immunohistochemistry (IHC) showed that most HDACs are expressed primarily in neurons, whereas expression in other cell types is isoform-specific or limited in the rat brain. Regarding age-related changes in HDAC expression in rodent brain, global HDAC enzymatic activity in the hippocampus and frontal cortex of 18-month-old rats is higher than that in 3-month-old rats [24]. In disease models, concentrations of HDAC3 and 4 in whole brain hemispheres of 5xFAD mice, an AD model with amyloid-β deposition, are about 1.5 times higher than those in wild-type mice [25]. Expression of class I and II HDACs in the non-human primate (NHP) brain was reported by Yeh et al. in a study evaluating a radioligand for HDAC PET imaging [26]. In this report, they performed qualitative IHC analysis of the expression of 11 HDAC isoforms in brain regions including the nucleus accumbens, hippocampus, cortex, and cerebellum. In 2008, Lucio-Eterovic et al. assessed mRNA expression of HDACs in the human brain with quantitative real-time polymerase chain reaction as part of an investigation into HDAC expression levels in gliomas [27]. In normal brain tissue, expression of class I HDACs, HDAC6, and HDAC7 is relatively low, whereas HDAC4, 5, 9, 10, and 11 show higher expression. Anderson et al. determined HDAC expression in human brain with multiple reaction monitoring mass spectrometry [25]. Concentrations of HDAC1 + 2, 5, and 6 were reported as 1.10, 0.083, and 0.106 pmol/mg tissue protein, respectively, in the frontal cortex of aged controls. In 2016, Wey et al. assessed expression levels of HDAC1, 2, 3, and 6 in human brains diagnosed with no neuropathological abnormalities as part of a clinical study of an HDAC PET ligand [28]. The amounts of HDAC2 and 3 are significantly higher in the superior frontal gyrus relative to the corpus callosum. The expression levels of HDAC2, 3, and 6 are comparable in the superior frontal gyrus (0.12–0.16 pmol/mg total protein), whereas that of HDAC1 is obviously higher than others (1.7 pmol/mg total protein). In brains with neurodegenerative disease, Ding et al. reported that compared with aged controls, the expression of HDAC6 in the cerebral cortex and hippocampus of AD patients is increased by 52% and 91%, respectively [29]. In a study by Anderson et al. in 2015, the concentrations of HDAC1 + 2, 5, and 6 in the frontal cortex of AD patients were reported to be 0.7, 1.5, and 1.3 times those of aged controls [25]. Whitehouse et al. investigated the distribution and intensity of HDAC4, 5, and 6 in FTLD with IHC [30]. HDAC4 and 6 show higher immunoreactivity in the dentate gyrus in FTLD cases, especially in FTLD-tau Picks, compared with controls, and the difference in HDAC6 was more prominent. No changes were observed for HDAC5 between FTLD and controls.

## 3. HDAC Inhibitors

**Figure 1:**
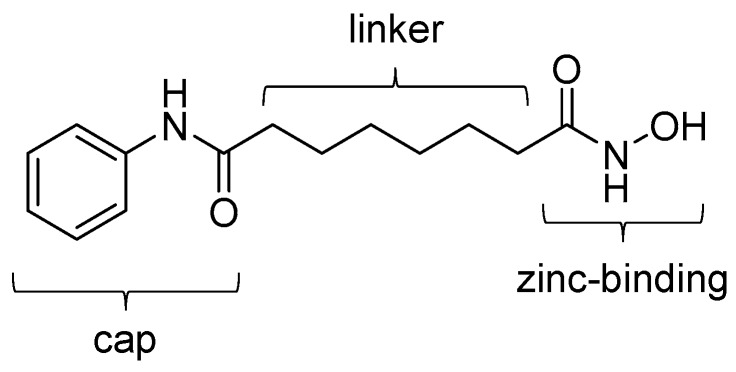
Chemical structure of SAHA. Three groups that constitute a typical HDAC inhibitor are indicated.

In the last two decades, many types of inhibitors against zinc-dependent HDACs have been developed for treatment of cancer and neurodegenerative disease [21,31,32,33]. Typical small molecule HDAC inhibitors can be classified into four classes: hydroxamic acids, alkanoic acids, cyclic peptides, and *ortho*-aminoanilides [19]. Following the discovery of the class I and II HDAC inhibitor, suberoylanilide hydroxamic acid (SAHA, also known as vorinostat) in 1996 [34], hydroxamic acid-based HDAC inhibitors compose the biggest compound library of the four classes. The chemical structure of HDAC inhibitors is generally composed of three motifs: a zinc-binding group that holds the zinc ion of HDAC, a cap group that interacts with the protein surface in the binding pocket, and a linker group that bridges the other two groups. For example, in SAHA, the zinc-binding, cap, and linker groups are *N*-phenylformamide, hydroxamic acid, and hexane groups, respectively (Figure 1). SAHA is the first US Food and Drug Administration (FDA)-approved HDAC inhibitor for use in patients with cutaneous T-cell lymphoma. The anticancer effects of SAHA are thought to be caused by modulation of both gene expression and acetylation of proteins that regulate cell proliferation [35]. Besides SAHA, other HDAC inhibitors (e.g., panobinostat and belinostat) have been approved by the US FDA for the treatment of cutaneous T-cell lymphoma, peripheral T-cell lymphoma, or multiple myeloma [36,37].

HDAC inhibitors have shown promising results in preclinical studies using animal models of neurodegenerative diseases [33,38]. Ricobaraza et al. reported that sodium 4-phenylbutyrate treatment reverses spatial memory deficits in Tg2576 AD model mice without affecting β-amyloid levels [39]. They attributed this effect to both activation of gene transcription by histone deacetylation inhibition and normalization of tau hyperphosphorylation by glycogen synthase kinase 3β inhibition. Gardian et al. showed neuroprotective effects of phenylbutylate treatment in 1-methyl-4-phenyl-1,2,3,6-tetrahydropyridine (MPTP)-induced Parkinson’s disease model mice [40]. Following phenylbutylate administration, depletion of dopamine in the striatum and reduction in tyrosine hydroxylase-positive neurons in the substantia nigra are attenuated. In R6/2 Huntington’s disease model mice, SAHA treatment improves motor impairment in the mice as measured with the rotarod performance test [41]. 

Although HDAC inhibitors are expected to be promising therapeutic tools for neurodegenerative diseases as described above, they may have adverse events [42]. HDAC inhibitors have pleiotropic effects on the different types of cells in the CNS because they often inhibit multiple HDACs that deacetylate a wide variety of substrates, including histones, transcription factors, and cytoplasmic proteins [32]. For this reason, isoform-specific HDAC inhibitors are likely to broaden the application of HDAC-targeted therapy [19]. In addition, the in vivo target engagement studies of HDAC inhibitors using HDAC PET imaging techniques described in this review will help estimate the desired and undesired CNS effects of the drugs.

## 4. Radioligands for HDACs

Radiosynthesis and preclinical characteristics of reported HDAC radioligands are summarized in the following sections. In this review, the radioligands were classified into four groups according to their structural properties: SAHA-based, adamantane-conjugated hydroxamic acid-based, other carboxylic acid- and hydroxamic acid-based, and *ortho*-aminoanilide-based ligands. Radioligands intended to image HDACs not only in the brain but also in tumors are included.

### 4.1. SAHA-Based Ligands

**Scheme 1:**
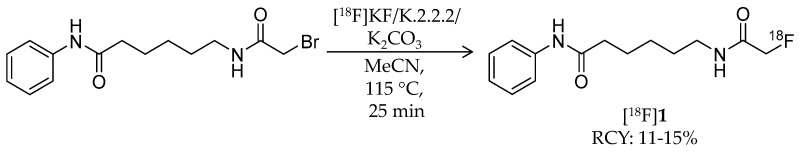
Radiosynthesis of [^18^F]**1**.

**Figure 2:**
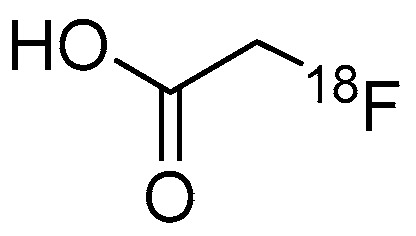
Chemical structure of [^18^F]FACE.

In 2006, radiosynthesis of the first radiolabeled ligand for imaging of HDAC expression and activity was reported [43]. Aiming to evaluate the HDAC expression and to estimate the effectiveness of SAHA, Mukhopadhyay et al. developed an ^18^F-labeled SAHA analogue, 6-([^18^F]fluoroacetamido)-1-hexanoicanilide ([^18^F]FAHA, [^18^F]**1**) (Table 1), which has an ^18^F-fluoroacetamido group as a substitute for a hydroxamic acid group. Although the fluoroacetamido group appeared to be a substrate for HDACs because of its similarity to acetyllysine, no characterization of its binding affinity and selectivity for HDACs was reported in the paper. Radiosynthesis of [^18^F]**1** was achieved by ^18^F-fluorination with a bromide precursor (Scheme 1). Using [^18^F]tetrabutylammonium fluoride ([^18^F]TBAF), reactions at 80 °C in several solvents such as acetonitrile (MeCN), tetrahydrofuran (THF), and dimethylsulfoxide (DMSO) for 20 min gave decay-corrected radiochemical yields of only 1.0–3.8% with an average of 1.8%, although non-radioactive fluorination with TBAF gave the desired product in >50% yield. The authors presumed that the low radiochemical yield was caused by loss of the product during a high-performance liquid chromatography (HPLC) purification step due to poor solubility of the product in the aqueous HPLC eluent. Meanwhile, reactions at 115 °C in MeCN for 25 min using [^18^F]KF/Kryptofix 2.2.2 gave [^18^F]**1** in a decay-corrected radiochemical yield of 11 ± 1.7% after HPLC purification. The radiochemical purity and the molar activity following this method were >99% and >74 GBq/μmol, respectively, at the end of synthesis. Nishii et al. performed in vivo characterization of [^18^F]**1** in rats using PET [44,45]. The uptake of [^18^F]**1** in the rat brain increased rapidly after intravenous (i.v.) injection and reached 0.44% injected dose (ID)/g at 5 min post-injection (p.i.). Brain uptake was significantly decreased (*p* < 0.01; *t*-test) by pre-treatment with SAHA [50 mg/kg; intraperitoneal (i.p.) administration] an hour before a PET scan [44]. Tumor uptake of [^18^F]**1** was also assessed using human breast carcinoma xenografts in rats, and as seen in the rat brain, the uptake was inhibited by pre-treatment with SAHA [45]. In 2009, Reid et al. reported a further biological study to evaluate the utility of [^18^F]**1** for PET imaging of HDAC activity [46]. They anticipated that HDAC may cleave [^18^F]**1** and generate a radiometabolite, [^18^F]fluoroacetate ([^18^F]FACE) (Figure 2). [^18^F]FACE crosses the blood-brain barrier (BBB) and is observed as a radiometabolite of PET tracers containing a 2-[^18^F]fluoroethyl group [47,48,49,50]. In vivo generation of [^18^F]FACE could cause high background radioactivity and complicate interpretation of PET data [48]. Consequently, they attempted to assess the in vivo biodistribution and metabolism of [^18^F]**1** as well as the biodistribution of independently synthesized [^18^F]FACE [51]. In baboons, almost all [^18^F]**1** was rapidly metabolized within 5 min in plasma, and regional differences in brain radioactivity uptake and kinetics were observed. In contrast, [^18^F]FACE showed gradual and uniform uptake in the same baboon brain, and the radioactivity peak was much smaller than that of [^18^F]**1**. Seven min after administration of [^18^F]**1** in rats, all of the radioactivity in the brain homogenates was in the form of [^18^F]FACE. Radioactivity accumulation in the bone suggested some in vivo defluorination in rats. Altogether, they concluded that the rapid metabolism and ability of the radiometabolite to penetrate the BBB complicated PET image analysis and that further studies about the interaction between [^18^F]**1** and HDACs were needed. Four years after this study, an international research group performed an [^18^F]**1** PET study in rhesus macaques combining computed tomography (CT) and magnetic resonance imaging (MRI) to quantify the rate of HDAC-mediated accumulation of [^18^F]**1** in the brain using an optimized pharmacokinetics model [26]. The pharmacokinetics model involved two blood plasma input functions for [^18^F]**1** and [^18^F]FACE and three tissue compartments. In their study, high substrate specificity of **1** for class IIa HDACs was demonstrated with an in vitro assay, which was consistent with a preceding study using trifluoroacetyl-lysine [52]. In addition to the multimodal imaging, they also assessed expression of individual HDACs and the deacetylation status of histones in the brain with IHC, and confirmed their heterogeneity in different brain structures and cell types. Quantitative analysis of PET/CT/MRI showed high accumulation of [^18^F]**1** in the nucleus accumbens and cerebellum in which high expression levels of class IIa HDACs such as HDAC 4 and 5 are observed. These results indicated that [^18^F]**1** accumulation in macaque brains depends on class IIa HDAC expression and activity, and consequently, [^18^F]**1** PET could be used for pharmacodynamics studies of class IIa HDAC inhibitors in the brain. Recently, the utility of [^18^F]**1** for PET imaging of lung cancer was evaluated using small animal PET/CT and a mouse model [53]. HDACs are also expected to be promising therapeutic targets in lung cancer [54]. A/J mice treated with 4-(methylnitrosamino)-1-(3-pyridyl)-1-butanone, a tobacco-specific precarcinogen, were used as lung cancer model mice [55]. In vivo PET imaging showed significant [^18^F]**1** uptake in lung tumors with a >2.0 tumor/nontumor ratio, which was slightly higher than those of [^18^F]fluorodeoxyglucose and [^18^F]nifene, an α4β2 nicotinic receptor radioligand [56].

**Scheme 2:**
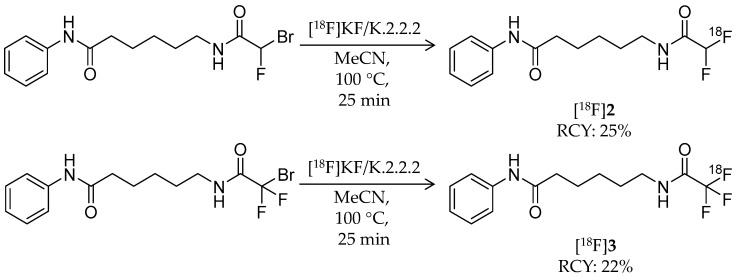
Radiosynthesis of [^18^F]**2** and [^18^F]**3**.

In 2015, two novel ^18^F-labeled successors to [^18^F]**1** were reported [57]. Bonimi et al. developed 6-(difluoroacetamido)-1-hexanoicanilide (DFAHA, [^18^F]**2**) and 6-(trifluoroacetamido)-1-hexanoicanilide (TFAHA, [^18^F]**3**) with improved selectivity and substrate efficiency for class IIa HDACs, which have important roles in the brain development and function (Table 1) [61]. These ligands were rationally designed in accordance with previous reports describing the substrate specificity of HDACs [52,62]. Non-radiolabeled **2** and **3** were synthesized from 6-amino-1-hexanoicanilide by reactions with corresponding di/trifluoroacetic anhydride. Radiosynthesis of [^18^F]**2** and [^18^F]**3** was achieved in a similar manner as that of [^18^F]**1** (Scheme 2). For [^18^F]**2** radiosynthesis, a bromofluoro precursor in MeCN was added to dried [^18^F]KF/K.2.2.2 and then stirred at 100 °C for 20 min. HPLC purification gave the desired product with a decay-corrected radiochemical yield of 25%, >95% radiochemical purity, and molar activity of 60–70 GBq/μmol. For [^18^F]**3** radiosynthesis, the same procedure except ^18^F-fluorination with additional heating at 110 °C for 25 min was performed to give the desired product with a decay-corrected radiochemical yield of 22%, >95% radiochemical purity, and molar activity of 70–80 GBq/μmol. In vitro comparison of substrate affinity of **1**, **2**, and **3** demonstrated that substitution of a fluorine atom for the hydrogen atom increased k_cat_ values for class IIa HDACs and decreased *v*_max_ values for class I HDAC8. In dynamic PET/CT imaging, high accumulation of [^18^F]**2** and [^18^F]**3** was observed in rat brain regions including the cerebellum, nucleus accumbens, and hippocampus, where class IIa HDACs are highly expressed. The brain radioactivity of [^18^F]**3** was higher than that of [^18^F]**2**, and it was significantly decreased by pre-treatment with SAHA (100 mg/kg; i.p. 30 min before [^18^F]**3** i.v.). These results suggested that [^18^F]**3** is a more suitable substrate-based radioligand to image the expression and activity of class IIa HDACs in the brain.

**Scheme 3:**
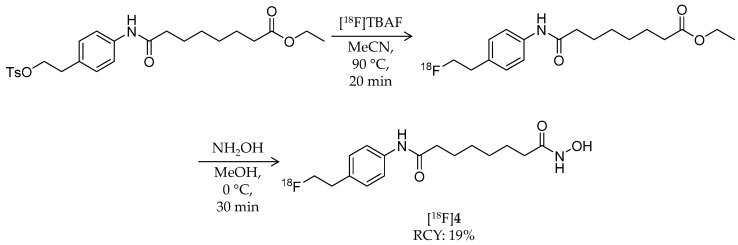
Radiosynthesis of [^18^F]**4**.

In 2011, Zeglis et al. reported the synthesis and evaluation of a hydroxamic acid-based HDAC radioligand, *N*^1^-(4-(2-fluoroethyl)phenyl)-*N*^8^-hydroxyoctanediamide (FESAHA, [^18^F]**4**) (Table 1) [58]. Unlike [^18^F]**1** that could be an HDAC substrate, [^18^F]**4** was expected to image the HDAC expression itself. [^18^F]**4** was radiosynthesized from a tosylate precursor with a one-pot, two-step reaction (Scheme 3). The precursor was ^18^F-fluorinated by a reaction with [^18^F]TBAF in MeCN at 90 °C for 20 min to obtain an ^18^F-intermediate, and then the solvent was removed by heating at 90 °C under a slow stream of argon. After the reaction vial was cooled to 0 °C, a solution of hydroxylamine in methanol was added and reacted for 30 min to convert the ethylester group into a hydroxamic acid group. HPLC purification and formulation gave the desired product in 19 ± 9% decay-corrected radiochemical yield and >99% radiochemical purity. The molar activity was about 2.3 GBq/μmol, which was relatively low for an ^18^F-labeled ligand. To ^18^F-radiolabel SAHA, they positioned a fluoroethyl group at the para position of the aniline ring. In silico modeling suggested that the modified structure would bind to the active site of an HDAC-like protein [63]. Furthermore, in vitro HDAC inhibition assays confirmed its submicromolar IC_50_ values ranging from 3 nM (HDAC6) to 474 nM (HDAC9) except 1.8 μM for HDAC4. Cell proliferation studies revealed identical cytostatic effects for **4** and SAHA against the prostate cancer cell lines, LNCaP and PC-3, both of which have increased HDAC expression [35,64]. A biodistribution study of [^18^F]**4** was performed in mice bearing LNCaP xenografts. Tumor uptake was 2.8 ± 0.3% ID/g at 30 min p.i. and decreased with time. Brain uptake was 1.0% ID/g or less over the course of 120 min p.i. Furthermore, significant radioactivity accumulation was observed in the bone (9.6 ± 2.2% ID/g at 30 min p.i.), suggesting substantial in vivo defluorination of [^18^F]**4**, which was confirmed by small animal PET imaging. Altogether, the radiolabeling position of [^18^F]**4** has little effect on its HDAC inhibition properties, but as a PET ligand, structural optimization is needed due to its metabolic instability.

**Scheme 4:**
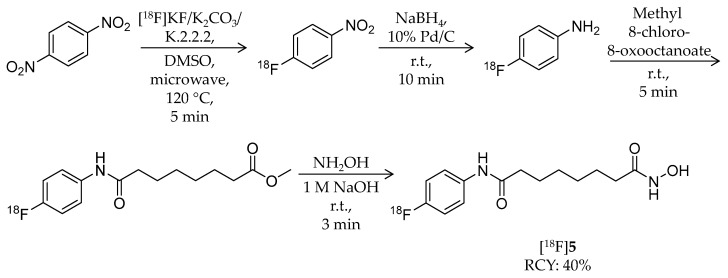
Radiosynthesis of [^18^F]**5**.

The same year, Hendricks et al. reported a close analogue of SAHA, *N*-hydroxy-*N′*-(4-fluoro-phenyl)octanediamide (*p*-fluoro SAHA, [^18^F]**5**), for characterization of SAHA as a cancer drug (Table 1) [59]. Non-radiolabeled *p*-fluoro SAHA was synthesized from 4-fluoroaniline by following reported strategies [65]. For radiosynthesis of [^18^F]**5**, 1,4-dinitrobenzene was used as a starting reagent (Scheme 4). The first intermediate, [^18^F]**1**-fluoro-4-nitrobenzene, was obtained by microwave heating of dinitrobenzene and [^18^F]KF/K_2_CO_3_/K.2.2.2 in DMSO at 120 °C for 5 min. A nitro group of the intermediate was reduced by NaBH_4_ and Pd/C to obtain [^18^F]**4**-fluoroaniline, and then it was isolated by solid phase extraction (SPE). Methyl 8-chloro-8-oxooctanoate was added, and the mixture was stirred at room temperature for 5 min. Finally, 50% hydroxylamine and 1 M NaOH in methanol was added and reacted for 3 min to convert the methyl ester group into a hydroxamic acid group. HPLC purification gave the desired product with 39.5 ± 6.0% decay-corrected radiochemical yield and 97.0 ± 4.7% radiochemical purity. **5** has identical inhibitory effects as SAHA against HDAC1, 2, 3, and 6. They determined the tissue distribution of [^18^F]**5** in mice. After i.v. injection of the radioligand, the highest radioactivity was observed in the kidney, liver, and blood, whereas brain uptake was the lowest. Bone uptake of radioactivity was not noticeable. They next assessed its accumulation in tumors using a mouse with a human ovarian cancer xenograft and small animal PET/CT. Tumor accumulation of [^18^F]**5** was observed in a time-dependent manner, and it decreased by 23% on pre-treatment with SAHA.

**Scheme 5:**
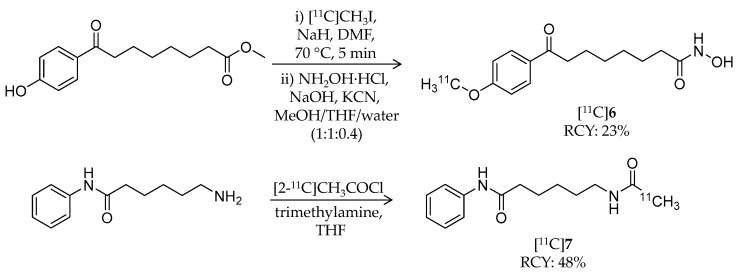
Radiosynthesis of [^11^C]**6** and [^11^C]**7**.

In 2013, Seo et al. reported radiosynthesis and biological evaluation of two ^11^C-labeled SAHA-based ligands ([^11^C]**6** and [^11^C]**7**) for HDAC imaging in the brain (Table 1) [60]. [^11^C]**6** was synthesized from a hydroxyl precursor by a two-step reaction of ^11^C-methylation and conversion of a methylester group into a hydroxamic acid group (Scheme 5). A mixture of the precursor, [^11^C]CH_3_I, and sodium hydride in DMSO was heated to 70 °C for 5 min, and then hydroxylamine hydrochloride was added. The authors used potassium cyanide as a base in the second step instead of sodium hydroxide because of the slow reaction rate and by-product formation [66]. HPLC purification gave the desired product in 23 ± 3% decay-corrected radiochemical yield (calculated from [^11^C]CH_3_I) and >99% radiochemical purity. The molar activity was 10.4 ± 1.9 GBq/μmol at the time of use. Synthesis of [^11^C]**7**, an analogue of **1**, was achieved using [2-^11^C]CH_3_COCl [67] (Scheme 5). Distillation of [2-^11^C]CH_3_COCl into a mixture of the precursor and trimethylamine in THF resulted in the formation of [^11^C]**7**. HPLC purification gave the desired product in 48 ± 7% decay-corrected radiochemical yield and >99% radiochemical purity. The molar activity was 6.0 ± 1.4 GBq/μmol at the time of use. Using PET, organ uptake and clearance of [^11^C]**6** and [^11^C]**7** in baboons were assessed over a 90-min period after i.v. injection. Arterial blood plasma was also collected, and the fraction of intact radioligand was measured with HPLC at different time points during the PET scan. [^11^C]**6** showed poor brain uptake in baboons, and the distribution pattern was homogeneous in different brain regions. Furthermore, no difference was observed in time-activity curves in peripheral organs between the presence and absence of SAHA pre-treatment, suggesting that no specific binding of [^11^C]**6** occurs in these organs. Contrary to a prediction by the authors, brain uptake of [^11^C]**7** was also poor even though the chemical structure of [^11^C]**7** closely resembles that of [^18^F]**1**. They confirmed that the unchanged [^11^C]**7** fraction in plasma at 10 min was higher than [^18^F]**1** (10% versus 1%), and therefore, the difference was not due to a metabolic difference. Thus, substitution of a hydrogen atom for a fluorine atom affected brain uptake and substrate specificity for HDACs more than expected. In summary, these radioligands are not suitable for imaging HDACs in the brain.

### 4.2. Adamantane-Conjugated Ligands

**Scheme 6:**
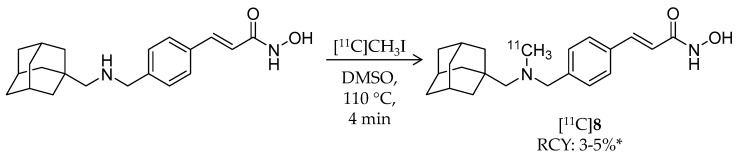
Radiosynthesis of [^11^C]**8**. * Non-decay corrected.

**Figure 3:**
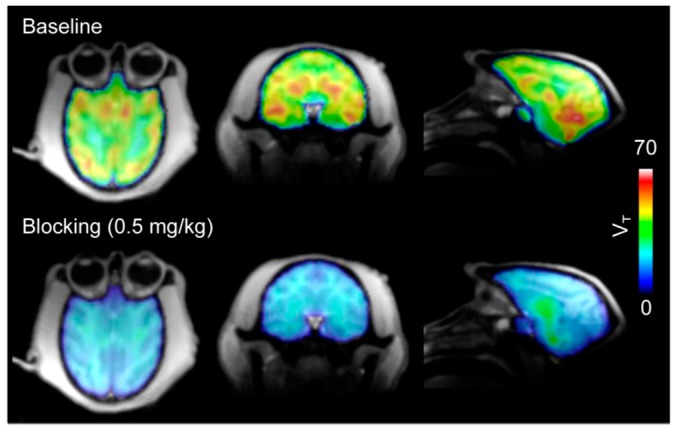
The total volume of distribution (*V*_T_) images from [^11^C]**8** PET in baboon brains. Robust brain uptake (**top**) of [^11^C]**8** was decreased by pre-treatment with unlabeled **8** (**bottom**). (Reproduced from Wang et al. J Med Chem; published by The American Chemical Society, 2014 [68]).

In 2014, the first adamantane-conjugated HDAC imaging radioligand was reported by Hooker et al. at Massachusetts General Hospital [68]. The adamantane “lipophilic bullet” is often conjugated to drugs to improve their brain penetrance [69,70,71], and adamantane-based hydroxamates are highly potent HDAC inhibitors [72]. Based on prior reports, Wang et al. synthesized (*E*)-3-(4-((((3r,5r,7r)-adamantan-1-ylmethyl)([^11^C]methyl)amino)methyl)phenyl)-*N*-hydroxyacrylamide ([^11^C]Martinostat, [^11^C]**8**) (Scheme 6) as a radioligand for quantification of the density of HDACs in the CNS and major peripheral organs (Table 2). The radiosynthesis of [^11^C]**8** was achieved by ^11^C-methylation of a desmethylated precursor. [^11^C]CH_3_I was trapped in a solution of the precursor in DMSO, and the solution was heated to 110 °C for 4 min. HPLC purification gave the desired product in 3–5% non-decay-corrected radiochemical yield (calculated from trapped [^11^C]CH_3_I) and ≥95% radiochemical purity with a molar activity of 37 ± 7.4 GBq/μmol. The log *D* value of [^11^C]**8** was 2.03. In vitro inhibitory activities of **8** against HDACs were measured using recombinant human enzymes. Low IC_50_ values were observed for HDAC1 (0.3 nM), 2 (2.0 nM), 3 (0.6 nM), and 6 (4.1 nM), moderate values were observed for HDAC4 (1970 nM) and 5 (352 nM), and high values were observed for HDAC7 (>20,000), 8 (>15,000), and 9 (>15,000). An in vitro radioligand displacement assay with four zinc-dependent enzymes and 80 additional non-HDAC and Zn-dependent CNS targets demonstrated that the dopamine transporter was a potential off-target binding site of [^11^C]**8**; however, binding of 2-β-carbomethoxy-3β-(4-fluorophenyl)-[*N*-^11^C-methyl]tropane, a dopamine transporter radioligand [73], in rat brains was not inhibited by **8**. In vitro autoradiography (ARG) confirmed specific binding of [^11^C]**8** in rat brain sections, and the binding was completely inhibited in the presence of excess SAHA. The authors assessed brain uptake of [^11^C]**8** with PET imaging using both rats and baboons. In rat brains, radioactivity accumulation was observed after i.v. injection of [^11^C]**8**, and accumulation decreased following pre-treatment of unlabeled **8** in a dose-dependent manner. Furthermore, brain uptake of [^11^C]**8** was not altered by pre-treatment with the P-glycoprotein (P-gp) inhibitor cyclosporine A (25 mg/kg, 30 min before administration of radioligand), suggesting that [^11^C]**8** is not a substrate of P-gp. Metabolism of [^11^C]**8** in rat brains was not noticeable. To analyze baboon PET data for [^11^C]**8**, a two-tissue compartmental model was used with a metabolite-corrected plasma time-activity curve. Plasma radioactivity was rapidly cleared after i.v. injection of [^11^C]**8**, although about 40% unchanged fraction still remained in plasma 30 min post-injection. As in rats, high brain uptake of [^11^C]**8** was observed in baboons, and uptake decreased following pre-treatment with unlabeled **8** (Figure 3). Following these promising results, the same group reported in vivo target engagement studies of a subset of HDAC inhibitors (5 hydroxamates, 4 *ortho*-aminoanilides, and 1 short-chain fatty acid) using [^11^C]**8** PET imaging in the rat brain [74]. Small molecule HDAC inhibitors modulate CNSdisease-related behaviors in rodents [13,75]; however, direct evidence of target engagement in the brain has not been demonstrated. In this study, they found that i.v. pre-treatment with hydroxamates containing heterocyclic capping groups [76] 3–10 min before [^11^C]**8** administration resulted in 20–40% blockage of [^11^C]**8** binding in the brain. Meanwhile, i.p. pre-treatment with *ortho*-aminoanilides [75,77] resulted in limited blockage of [^11^C]**8** binding. To test whether adamantane could improve brain penetrance of *ortho*-aminoanilides like hydroxamates, they synthesized an adamantane-conjugated *ortho*-aminoanilide named CN147. This novel compound showed 25% blockage of [^11^C]**8** binding, suggesting that it enhanced HDAC engagement in the brain. Furthermore, an antidepressant-like effect of CN147 was confirmed with a rat behavioral test. These results suggested the utility of [^11^C]**8** PET as a tool for investigating in vivo target engagement of HDAC inhibitors. Kinetic analysis with arterial plasma sampling of [^11^C]**8** in different brain regions of the baboon was also reported [78].

**Scheme 7:**
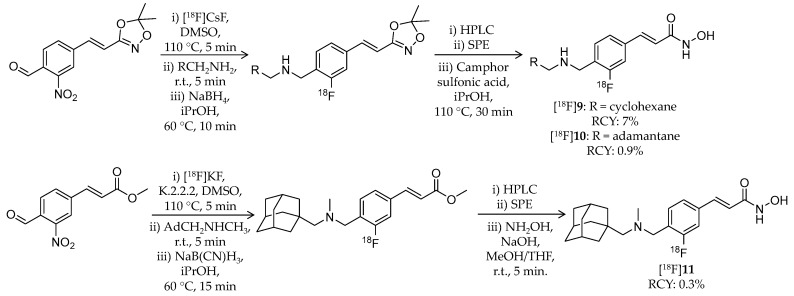
Radiosynthesis of [^18^F]**9**–**11**.

Besides [^11^C]**8**, Strebl et al., from the same group at Massachusetts General Hospital, developed ^18^F-labeled adamantane- or cyclohexane-conjugated ligands in 2016 [79]. The three novel compounds have a similar structure to [^11^C]**8** and a fluorine atom bound directly to a benzene ring ([^18^F]**9**–**11**) (Scheme 7, Table 2). [^18^F]**9** and [^18^F]**10** were synthesized using the same precursor, 3-(4-formyl-2-nitrostyryl)-5,5-dimethyl-1,4,2-dioxazole. A mixture of the precursor, Cs^18^F, and DMSO was heated to 110 °C for 5 min followed by transfer into another vial containing the corresponding amine and stirring at room temperature for 5 min. Then a saturated solution of sodium borohydride in isopropanol was added. After stirring at 60 °C for 10 min, the mixture was purified by semi-preparative HPLC. The isolated intermediate was passed through a C18 SPE cartridge and eluted with isopropanol. Camphor sulfonic acid was added, and the mixture was heated to 110 °C for 30 min. HPLC purification gave the desired products in 7% and 0.9% decay-corrected radiochemical yield for [^18^F]**9** and [^18^F]**10**, respectively. [^18^F]**11** was synthesized using methyl 4-formyl-3-nitrocinnamate as the precursor. A mixture of the precursor, [^18^F]KF/K_2_CO_3_/K.2.2.2, and DMSO was heated to 110 °C for 5 min. After SPE with a C18 cartridge, (1-adamantylmethyl)-*N*-methylamine was introduced. The mixture was reacted with NaB(CN)H_3_ and then purified with HPLC. Finally, a methyl ester group was converted to a hydroxamic acid group using hydroxylamine and sodium hydroxide.

HPLC purification gave the desired product in 0.3% decay-corrected radiochemical yield. All three compounds showed high affinity for HDAC1, 2, 3, and 6 (IC_50_ values: <20 nM). Brain kinetics of the radioligands was assessed with PET imaging in rats and baboons. In rats, all radioligands showed rapid brain uptake after injection, followed by a gradual decrease in radioactivity over the 2-h scan. By pre-treatment with unlabeled **8**, washout rates of the radioligands from the brain were increased, and brain radioactivity decreased to about 50% of peak within 2 h. Meanwhile, PET imaging in baboons revealed differences between the radioligands in brain kinetics. A brain standardized uptake value (SUV) from 30 to 60 min of [^18^F]**9**, the cyclohexyl compound, was only 0.57, whereas those of [^18^F]**10** and [^18^F]**11** reached 1.22 and 1.80, respectively, suggested that the adamantane group enhanced the BBB permeability. On the other hand, the brain uptake of [^18^F]**11** was slightly lower than that of [^11^C]**8** although they are close analogues. Furthermore, the regional SUV of ^18^F-labeled ligands in baboon brains were compared to that of [^11^C]**8**.

Although all three radioligands showed significant correlation with [^11^C]**8**, correlation between [^18^F]**11** and [^11^C]**8** was the highest. Taken together, [^18^F]**11** exhibited comparable properties to [^11^C]**8**, such as HDAC binding and brain uptake, although further optimization of radiosynthesis is needed.

**Scheme 8:**
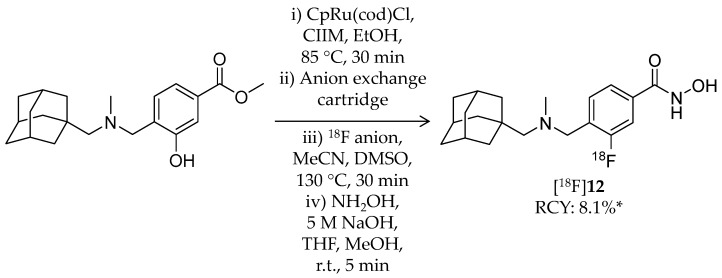
Radiosynthesis of [^18^F]**12**. * Non-decay corrected.

Very recently, Strebl et al. reported synthesis and evaluation of an ^18^F-labeled hydroxamic acid-based radioligand, [^18^F]Bavarostat ([^18^F]**12**, Scheme 8), with selectivity for HDAC6 (Table 2) [80]. HDAC6 is receiving increased attention as a therapeutic target for the neurodegenerative diseases [81]. [^18^F]**12** has a similar structure as [^18^F]**11** but without a vinyl group in the linker. Close approach of a bulky moiety to the zinc-binding group is one of the factors that achieve the selectivity [82]. Radiolabeling of [^18^F]**12** was achieved by a recently reported ^18^F-deoxyfluorination method [83,84,85]. First, a mixture of 4-((((adamantan-1-yl)methyl)(methyl)amino)methyl)-3-hydroxybenzoate, CpRu(cod)Cl, *N*,*N*-bis(2,6-diisopropyl)phenyl-2-chloroimidazolium chloride, and ethanol was heated to 85 °C for 30 min to form an *η*^6^ coordinated ruthenium–phenol complex. The resulting solution was passed through an ^18^F anion-bearing anion exchange cartridge, which was flushed with MeCN and DMSO. The elution was heated to 130 °C for 30 min, and then a hydroxylamine solution was added to convert a methyl ester group to a hydroxamic acid group. HPLC purification gave the desired product in 8.1% non-decay-corrected radiochemical yield with a molar activity of 148 GBq/μmol. Radiochemical purity was not stated in the published article. Regarding selectivity, IC_50_ values of **12**, determined using recombinant enzymes, were 0.06 μM for HDAC6 and >1 μM for other HDACs. **12** also showed selective deacetylation inhibition of α-tubulin, a substrate of HDAC6, in human induced pluripotent stem cell-derived neural progenitor cells. In vitro ARG using rat brain sections demonstrated specific binding of [^18^F]**12**, and the binding was blocked by the HDAC6 selective inhibitor, tubastatin A [82]. Brain kinetics of [^18^F]**12** was assessed with PET imaging in a baboon. The brain uptake of [^18^F]**12** reached an SUV of 3 immediately after injection and gradually decreased over a 120-min period. Pre-treatment with 1.0 mg/kg unlabeled **12** resulted in a substantial decrease in baboon brain uptake of [^18^F]**12**. Preliminary analysis showed a good correlation between SUV and total distribution volume (*V*_T_). In summary, a promising HDAC6 PET radioligand with BBB permeability was successfully developed, and further biological evaluation and optimization of radiosynthesis are awaited for a clinical study.

### 4.3. Other Carboxylic Acid- and Hydroxamic Acid-Based Ligands

**Scheme 9:**
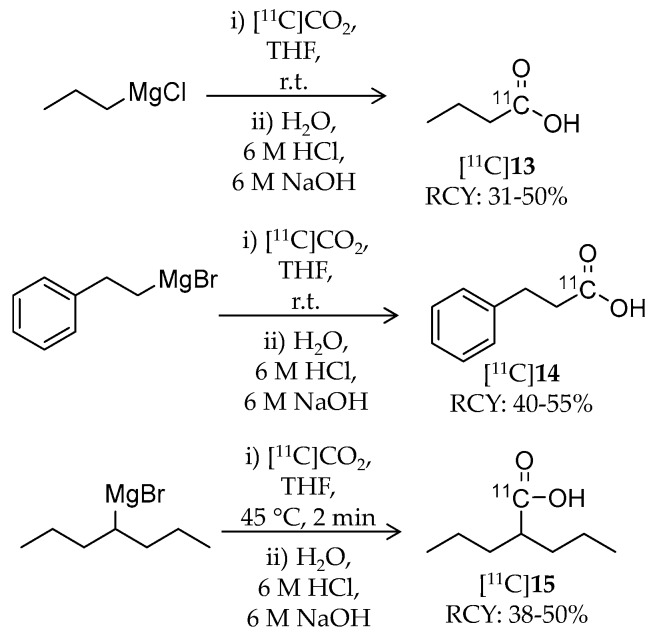
Radiosynthesis of [^11^C]**13**–**15**.

Pharmacokinetic evaluation of three ^11^C-labeled alkanoic acid-based HDAC inhibitors, *n*-butyric acid, 4-phenylbutylic acid, and valproic acid, was reported by Kim et al. in 2013 (Table 3) [86]. Generally, HDAC inhibitory activities of these alkanoic acids (IC_50_ in the μM range) are weaker than those of hydroxamic acid- or benzamide-based inhibitors [87]. Although these radioligands have been used to treat several diseases including CNS diseases, information about their distribution and pharmacokinetics in the brain was limited. The authors introduced carbon-11 at the carbonyl carbon of carboxylic acid by reaction of ^11^C-carbon dioxide and the respective Grignard reagents (Scheme 9). Briefly, [^11^C]CO_2_ was passed through a THF solution of the precursor. The reaction was quenched by water followed by sequential addition of 6 N hydrochloric acid and 6 N sodium hydroxide. For the synthesis of [^11^C]**15**, the reaction was performed with non-radioactive CO_2_ as a carrier with heating to 45 °C for 2 min. The products were purified with HPLC. Radiochemical yield (decay corrected at the end of cyclotron bombardment) and molar activity were 31–50% and 7.4–37 GBq/μmol for [^11^C]**13**, 40–55% and 7.4–30 GBq/μmol for [^11^C]**14**, and 38–50% for [^11^C]**15**, respectively (the molar activity of [^11^C]**15** was not mentioned). Radiochemical purities of all radioligands were greater than 98%. Whole-body pharmacokinetics of these radioligands was determined with PET imaging in baboons. Of the three radioligands, [^11^C]**15** was the most stable in baboon plasma (>90% intact ligand over 90 min). PET images demonstrated very low uptake of these radioligands in the baboon brain of less than 0.006% ID/cc, presumably due to their poor BBB penetrance. Interestingly, [^11^C]**15** showed relatively high heart uptake consistent with the cardiac side effects of **15** [88]. These findings obtained by the imaging studies can help in understanding therapeutic profiles of the inhibitors.

**Scheme 10:**
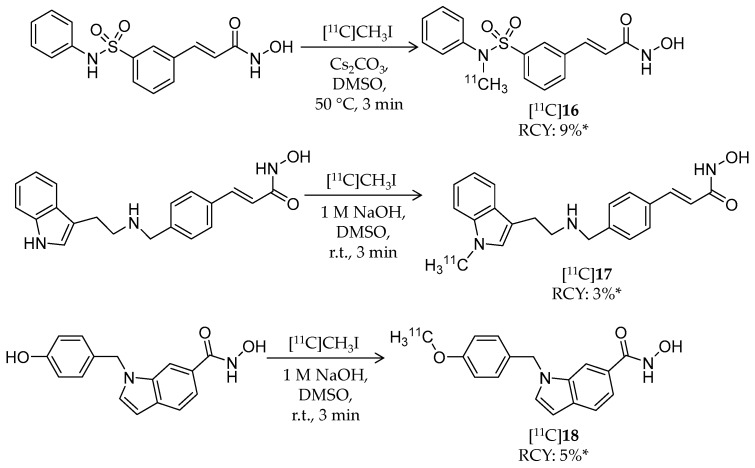
Radiosynthesis of [^11^C]**16**–**18**. * Non-decay corrected.

In 2014, Wang et al. reported the synthesis and evaluation of radiolabeled hydroxamic acid-based ligands for HDAC imaging [89]. Aiming to investigate the brain kinetics of hydroxamates, they selected three known HDAC inhibitors and modified their structures for ^11^C-labeling (Scheme 10, Table 3) [90,91,92]. 

Radiosynthesis of [^11^C]**16**–**18** was achieved by a reaction of precursors and [^11^C]CH_3_I in DMSO in the presence of base. Radiochemical yield (non-decay corrected, calculated from trapped [^11^C]CH_3_I) and molar activity were 9% and 29.6 ± 7.4 GBq/μmol for [^11^C]**16**, 3% and 33.3 ± 3.7 GBq/μmol for [^11^C]**17**, and 5% and 25.9 ± 7.4 GBq/μmol for [^11^C]**18**, respectively. Chemical and radiochemical purities were ≥95% for all radioligands. **16** and **17** showed low IC_50_ values against class I HDACs (HDAC1–3) and HDAC6, whereas **18** exhibited high HDAC8 selectivity with an IC_50_ value of 18.3 nM. The brain kinetics of all three radioligands was evaluated with PET imaging in rodents and NHPs. In rats, the radioligands exhibited poor brain uptake with less than 0.25% ID/cc over a 30-min p.i. period, and brain uptake was not changed by pre-treatment with 2 mg/kg unlabeled compound. Brain uptake in a baboon was also very limited. Altogether, these imaging results indicated that these radioligands are not suitable for in vivo HDAC imaging in the brain.

**Scheme 11:**
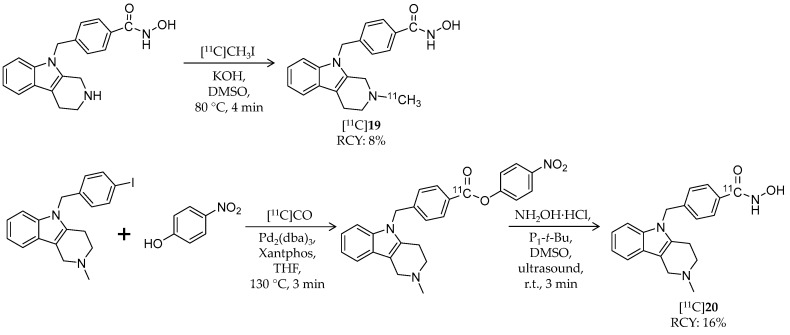
Radiosynthesis of [^11^C]**19** and [^11^C]**20**.

Lu et al. reported development of an HDAC6-selective hydroxamic acid-based radioligand [93]. [^11^C]**19** is a ^11^C-labeled analogue of tubastatin A, a selective HDAC6 inhibitor with a tetrahydro-γ-carboline structure (IC_50_: 15 nM for HDAC6; 1640 nM for HDAC1) (Scheme 11, Table 3) [82,94]. ^11^C-Methylation at the piperidine nitrogen atom was achieved using a desmethyl precursor and [^11^C]CH_3_I. [^11^C]CH_3_I was trapped in a solution of the precursor and KOH in DMSO, and the mixture was heated to 80 °C for 4 min. HPLC purification gave the desired product in 7.6% radiochemical yield from [^11^C]CO_2_ and >99% radiochemical purity. The molar activity was 96.2 GBq/μmol. IC_50_ values of **16** were 1.40 nM and 5180 nM for HDAC6 and HDAC1, respectively [94]. PET imaging with a rhesus monkey demonstrated limited radioactivity in the forebrain (SUV = 0.18) and cerebellum (SUV = 0.38). Furthermore, in rats pre-treated with a P-gp inhibitor 20 min before radioligand injection, [^11^C]**19** uptake in the forebrain (SUV = 0.44) and cerebellum (SUV = 0.48) was also low. The authors assumed this low BBB permeability was due to low lipophilicity of the compound (cLog*D* = 1.33). 

Recently, the same group explored ^11^C-labeling of tubastatin A at the carbonyl carbon of hydroxamic acid using [^11^C]carbon monoxide [95]. [^11^C]CO has the potential to radiolabel a wide variety of carbonyl-containing molecules [97], and this labeling method could be applied to other HDAC ligands with a hydroxamic acid group. Initially, the authors attempted to synthesize [^11^C]**20** in a one-step palladium-mediated reaction using the iodinated precursor, hydroxylamine, and [^11^C]CO; however, the decay-corrected radiochemical yield of [^11^C]**20** was less than 10% (calculated from [^11^C]CO_2_), and a carboxylic acid derivative was obtained as a major product. Then they tested a two-step reaction of ^11^C-methyl ester formation followed by conversion to a hydroxamate. The first step was achieved with a moderate radiochemical yield of 18.5 ± 6.2%, but the second step with hydroxylamine hydrochloride did not work. Through trial and error, they decided to investigate the use of a *p*-nitrophenyl ester, with greater susceptibility toward aminolysis, as a labeled intermediate (Scheme 11).

Using Pd_2_(dba)_3_ and Xantphos, [^11^C]CO insertion was successfully obtained with a radiochemical yield of 54.6 ± 8.0%. Furthermore, after investigation into the reaction condition, the second step with a phosphazene base gave the desired product in 16.1 ± 5.6% decay-corrected radiochemical yield with 8.2 GBq/μmol molar activity. Aiming to evaluate the versatility of this labeling method, three simple aryl iodides (e.g., 1-chloro-4-iodobenzene or 4-iodoanisole) were assessed. However, although corresponding *p*-nitrophenyl esters were obtained in good radiochemical yields, the second step reaction gave carboxylic acid derivatives. Taken together, [^11^C]**20** was labeled at the carbonyl carbon using [^11^C]CO, but application of the labeling method for the other hydroxamates required further optimization.

**Scheme 12:**
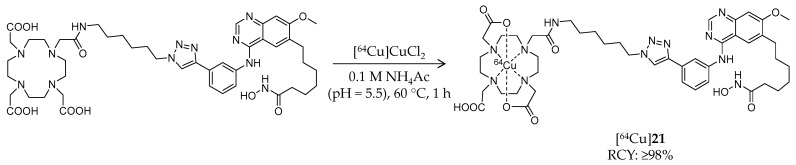
Radiosynthesis of [^64^Cu]**21**.

In addition to ^11^C- and ^18^F-labeled HDAC radioligands, radio-metal-labeled ligands have also been developed. In 2013, Meng et al. reported synthesis and biological evaluation of a ^64^Cu-labeled hydroxamic acid-based radioligand (Table 3) [96]. In this study, a chelator, 1,4,7,10-tetraazacyclododecane-1,4,7,10-tetraacetic acid, was conjugated to an HDAC inhibitor, CUDC-101 [98,99], with an aliphatic linker by Huisgen cycloaddition. [^64^Cu]**21** was labeled following a reaction of the precursor and [^64^Cu]CuCl_2_ in ammonium acetate buffer at 60 °C for 1 h (Scheme 12). HPLC purification gave the desired product in ≥98% radiochemical yield and radiochemical purity. The molar activity was estimated to 2.4–2.9 GBq/μmol. The IC_50_ value of **21** against class I and II HDACs was 94.47 ± 19.92 nM. An in vitro binding assay using MDA-MB-231, a breast cancer cell line with high HDAC expression [100], demonstrated dose-dependent inhibition of [^64^Cu]**21** cell uptake in the presence of CUDC-101. Subsequently, biodistribution of [^64^Cu]**21** in mice bearing an MD-MBA-231 xenograft was evaluated. PET imaging showed tumor uptake of radioactivity (1.20 ± 0.21, 2.16 ± 0.08, and 2.36 ± 0.31% ID/g at 2, 6, and 24 h p.i., respectively), and the uptake was reduced by half by coinjection of 20 mg/kg CUDC-101. The mice were sacrificed immediately after the PET scan and tissue radioactivity was measured. The biodistribution results demonstrated tumor/muscle and tumor/blood uptake ratios of 9.61 ± 1.54 and 4.44 ± 0.88, respectively. The brain uptake is not described in the paper probably because radioligands with a bulky motif such as metal chelators are generally not expected to cross the BBB. These results suggested the feasibility of HDAC imaging with radiometal-labeled ligands in cancer.

### 4.4. Ortho-Aminoanilide-Based Ligands

**Scheme 13:**
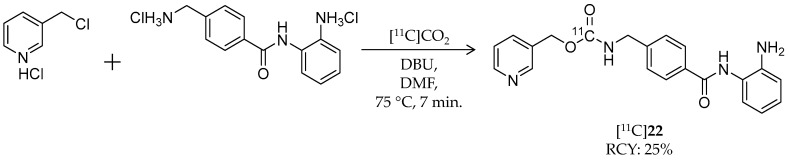
Radiosynthesis of [^11^C]**22**.

In 2010, Hooker et al. reported synthesis and evaluation of the first benzamide-based HDAC radioligand, [^11^C]MS-275 ([^11^C]**22**, Scheme 13), and characterized its pharmacokinetics in the brain (Table 4) [101]. **22** (IC_50_: ~300 nM for HDAC1; ~8 μM for HDAC3 [102]) is a potent, long-lasting, brain region-selective HDAC inhibitor that shows a dose-dependent increase in histone 3 acetylation in the rat brain [103]; however, no direct evidence was obtained for BBB permeability of **22**. They synthesized [^11^C]**22** by direct incorporation of [^11^C]CO_2_ into the carbamate carbon [104]. [^11^C]CO_2_ was trapped in a mixture of 3-picolyl chloride hydrochloride, 4-(aminomethyl)-*N*-(2-aminophenyl)-benzamide dihydrochloride, 1,8-diazabicyclo[5.4.0]undec-7-ene (DBU), and DMF, which was then heated to 75 °C for 7 min. HPLC purification gave the desired product in 25% decay-corrected radiochemical yield and >98% radiochemical purity. The molar activity was 100–229 GBq/μmol. Using [^11^C]**22**, they performed PET imaging studies in baboon and rat brains. In baboons, no radioactivity uptake was observed in the brain after i.v. injection of [^11^C]**22**. Pre-treatment with the P-gp substrate, verapamil (0.5 mg/kg, i.v., 5 min before [^11^C]**22** injection), had no influence on brain pharmacokinetics of [^11^C]**22**, suggesting that P-gp was not involved in [^11^C]**22** exclusion from the brain. Arterial plasma analysis demonstrated moderate metabolic stability of [^11^C]**22**; greater than 50% of radioactivity was derived from intact [^11^C]**22** at 90 min p.i. A further PET study in rats also confirmed poor brain uptake of [^11^C]**22**, and brain uptake was not altered by pre-treatment with unlabeled **19**. These results suggested that MS-275 may be not suitable for the treatment of neurodegenerative diseases targeting HDACs, but it is also likely to have minimal unwanted CNS effects in treatment of peripheral cancer.

**Scheme 14:**
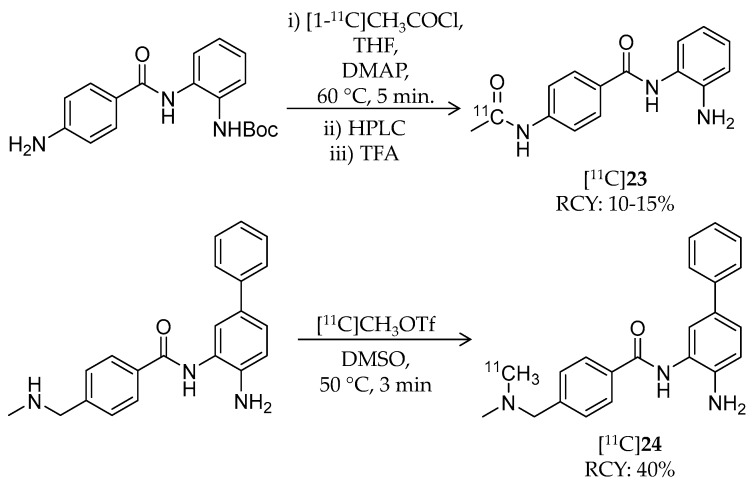
Radiosynthesis of [^11^C]**23** and [^11^C]**24**.

Seo et al. conducted PET imaging-guided systematic development of BBB-permeable HDAC inhibitors (Table 4) [105]. They repeated radiosynthesis, performed PET imaging in the baboon brain, and incorporated the imaging data into the compound design. For this study, several ^11^C-labeled benzamides were radiosynthesized using [^11^C]methyl iodide, [^11^C]methyl triflate, and [^11^C]acetyl chloride. Radiosynthesis of two representative benzamides, [^11^C]**23** and [^11^C]**24**, is shown in Scheme 14. **23** is the known HDAC inhibitor, CI-994, with class I HDAC selectivity (dissociation constant: 0.055 μM for HDAC1; 0.255 μM for HDAC2; 0.024 μM for HDAC3 [106]). For [^11^C]**23** radiosynthesis, [1-^11^C]CH_3_COCl, prepared from [^11^C]CO_2_, was added to a mixture of a precursor, 4-*N*,*N*-dimethylaminopyridine, and THF and heated to 60 °C for 5 min. After HPLC purification, the fraction containing the ^11^C-labeled intermediate was mixed with trifluoroacetic acid, followed by removal of the solvent *in vacuo*. The desired product was obtained, and the decay-corrected radiochemical yield and radiochemical purity were 10–15% and >95%, respectively. The molar activity was 200 MBq/μmol. For [^11^C]**24** radiosynthesis, [^11^C]CH_3_OTf was trapped in the precursor containing DMSO, and the mixture was heated to 50 °C for 3 min. HPLC purification gave the desired product in 40% decay-corrected radiochemical yield and 99% radiochemical purity. The molar activity was 185–666 GBq/μmol. IC_50_ values of **24** were 0.01 μM and 0.02 μM against HDAC1 and HDAC2, respectively. Initially, the authors assessed the BBB permeability of some compounds including [^11^C]**23**, but brain uptake in baboon was very limited. After image-guided structural optimization, *N*,*N*-dimethylamino derivatives such as [^11^C]**24** demonstrated improved brain uptake (~0.015% ID/cc at 5 min p.i.). [^11^C]**24** showed the highest area under the curve ratio of the brain versus plasma of 7.5. Furthermore, pre-treatment with unlabeled **24** (1 mg/kg) resulted in a decrease in *V*_T_ in the range of 13–25% compared with baseline in the cerebellum, thalamus, and temporal cortex. In this study, the polar surface area of less than 65 appeared to be a critical property for BBB permeability, and a key element that improved brain uptake of the benzamides was a benzylic amine.

### 4.5. First-in-Human PET Study

Findings in HDAC radioligand studies with NHPs are summarized in Table 5. Unfortunately, almost all HDAC radioligands showed poor BBB penetrance in PET studies with NHPs, probably due to their low lipophilicity or metabolic instability.

**Figure 4:**
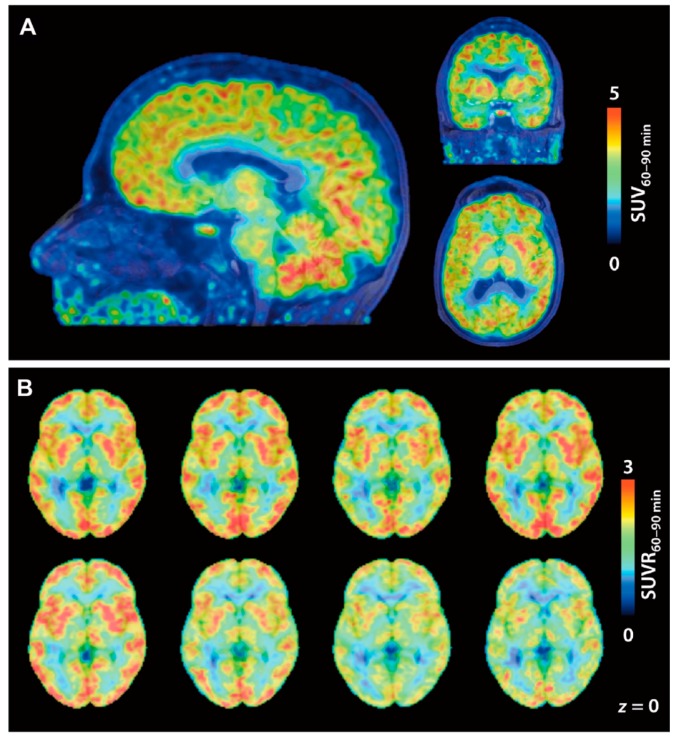
[^11^C]**8** PET images in a first-in-human study. (**A**) Brain SUV images averaged from 60 to 90 min p.i. of [^11^C]**8** (174 MBq). PET images are overlaid on MRI; (**B**) [^11^C]**8** SUVR images of eight individual subjects. SUV_60–90 min_ was normalized to white matter as a reference. (Reproduced with permission from Wey et al. Sci Transl Med; The American Association for the Advancement of Science, 2016 [28]).

Incidentally, in vivo P-gp blocking studies in rodents or NHPs were performed for some radioligands but showed no difference in brain uptake compared to baseline, suggesting that they are not P-gp substrates [68,93,101]. [^18^F]**1**, the first HDAC-targeting radioligand, showed a significant accumulation of radioactivity in the rhesus macaque brain, and substrate specificity analysis and immunohistochemical analysis for HDAC isoforms confirmed that these results reflect the level of class IIa HDACs in the brain [26]. However, quantitative analysis required consideration of the influence of brain uptake of a major radiometabolite, [^18^F]FACE. After this report in 2013, we could not find publications on clinical studies of [^18^F]**1**. Adamantane-conjugated hydroxamic acid-based HDAC radioligands tended to show preferable brain uptake in PET studies with NHPs, and especially, the study of kinetic analysis of [^11^C]**8** is well advanced [68,79,80]. An in vivo blocking experiment using [^11^C]**8** PET is also expected to be a tool to estimate brain penetrance of HDAC inhibitors for CNS diseases [74]. For ^18^F-labeled adamantane-conjugated ligands, further preclinical studies including metabolism analysis and brain kinetics analysis in NHPs are awaited. Following the promising preclinical studies, Wey and Gilbert et al. reported the first-in-human evaluation of [^11^C]**8** PET in 2016 [28]. They conducted [^11^C]**8** PET imaging on eight healthy volunteers (four females and four males; 28.6 ± 7.6 years old). [^11^C]**8** showed rapid brain uptake after injection followed by a slight increase in brain radioactivity during 90 min p.i. Cortical SUV from 60 to 90 min was twice that of white matter, and consequently, white matter was selected as a reference region to determine SUV_60–90 min_ ratios (SUVRs) (Figure 4). The lowest SUVRs were observed in the hippocampus and amygdala (around 1.5), and the highest SUVRs were observed in the putamen and cerebellum (around 2). *V*_T_ values determined using the metabolite-corrected arterial plasma as input fraction were stable beyond 50 min p.i., whereas information about the degree of unchanged radioligand in plasma was not described in the paper. The regional SUV_60–90 min_ correlated well with the *V*_T_ values (*r* = 0.98, *p* < 0.0001); therefore, SUV_60–90 min_ may be useful for quantification of [^11^C]**8** in future studies without arterial blood sampling. Test-retest scans (3 h apart) in three subjects showed less than 3% variability in SUV_60–90 min_. Western blotting analysis of postmortem brain tissues, which were obtained independently from the imaging study, demonstrated that amounts of HDAC2 and 3 in the superior frontal gyrus (gray matter) were significantly higher than those in the corpus callosum (white matter), whereas differences in HDAC1 and 6 amounts were not significant between the superior frontal gyrus and corpus callosum. In further biochemical profiling using a thermal shift assay, [^11^C]**8** was confirmed to selectively bind to HDAC1, 2, and 3. Although more research is needed to evaluate age-related and disease-related changes in HDAC expression, this study provided a critical foundation for quantification of epigenetic activity in the human brain.

## 5. Conclusions

Epigenetic abnormalities in the brain have been attracting more attention as therapeutic targets for neurodegenerative diseases. In this regard, non-invasive imaging techniques that can quantify the expression and/or activity of epigenetics-related enzymes in the living brain may deepen our understanding of the role of epigenetics in neurodegeneration and also help in development of new treatments; nevertheless, no clinically applicable technique currently exists. Although several types of epigenetic enzymes are expressed, development of PET imaging ligands for HDACs is particularly advanced at present. In this review, we summarized the radiosynthesis and preclinical characteristics of dozens of potential HDAC PET ligands developed in this decade to identify the achievements and challenges in the field. 

Brain uptake of just under 20 HDAC radioligands has already been assessed in NHPs with PET (Table 5); however, few of these radioligands readily enter the brain. Some radioligands showed low brain uptake in NHPs, although they had a preferable molecular weight and lipophilicity to cross the BBB by passive transport [107]; therefore, investigation into the background of the poor BBB permeability could help optimize radioligands for HDAC imaging in the brain. Besides the radioligands with binding affinity for multiple HDACs, the development of isoform-specific ligands is also awaited because disease-related changes in HDAC expression may be isoform specific. In this regard, evaluation of in vivo selectivity of radioligands (e.g., in vivo blocking assays using selective HDAC inhibitors and comparison of ex vivo ARG and immunohistochemical staining in rodent brain sections) is important in addition to in vitro HDAC inhibition assays. Moreover, PET imaging studies with disease model animals could reveal the time course of pathological changes and cognitive and behavioral manifestations by performing multiple longitudinal scans in the same subject. At the moment, the adamantane-conjugated radioligand, [^11^C]**8**, seems to be the most promising radioligand to image HDACs in the human brain. Clinical studies using [^11^C]**8** to assess age-related and disease-related changes in brain HDACs could provide valuable information about the roles of HDACs in neurodegenerative diseases.

## Figures and Tables

**Table 1:** Physiochemical properties of radiolabeled SAHA-based HDAC ligands.

Compound	MW	Log *D*	RCY	Target	IC_50_ for HDACs	In Vivo Properties in Rodents	Reference
[^18^F]**1**	265.32	1.39	11–15%	Class IIa	–	Brain uptake: 0.44 and 0.40% ID/g at 5 and 60 min, respectively (rat)	[26,43,44,46]
[^18^F]**2**	283.31	–	25%	Class IIa	–	Brain uptake: SUVs were around 1 in various brain regions during the 60-min scan (rat)	[57]
[^18^F]**3**	301.30	–	22%	Class IIa	–	Brain uptake: SUVs were around 1 in various brain regions during the 60-min scan (rat)	[57]
[^18^F]**4**	309.37	1.01	19%	Non-selective	HDAC1: 56 nM HDAC5: 67 nM HDAC6: 3 nM HDAC7: 85 nM	Brain uptake: 1.0, 0.7, and 0.4% ID/g at 30, 60, and 120 min, respectively Defluorination: 9.6, 11.1, and 13.4% ID/g bone uptake at 30, 60, and 120 min, respectively (tumor-bearing mouse)	[58]
[^18^F]**5**	281.32	–	40%	HDAC1–3/6	HDAC1: 9.0 nM HDAC2: 13 nM HDAC3: 24 nM HDAC6: 50 nM	Brain uptake: Very limited in biodistribution study (mouse/tumor-bearing mouse)	[59]
[^11^C]**6**	278.34	0.5	23%	–	–	–	[60]
[^11^C]**7**	247.33	1.7 ^1^	48%	–	–	–	[60]

^1^ Clog *P*. MW: molecular weight; RCY: radiochemical yield (decay corrected); SUV: standardized uptake value; ID: injected dose.

**Table 2:** Physiochemical properties of radiolabeled adamantane-conjugated HDAC ligands.

Compound	MW	Log *D*	RCY	Target	IC_50_ for HDACs	In Vivo Properties in Rodents	Reference
[^11^C]**8**	353.49	2.03	3–5% ^2^	Class I/IIb	HDAC1: 0.3 nM HDAC2: 2.0 nM HDAC3: 0.6 nM HDAC6: 4.1 nM	Brain uptake: Around 0.5% ID/cc during the 60-min scan Metabolism: Radiometabolites in the brain were limited (rat)	[28,68,74,78]
[^18^F]**9**	305.38	3.07 ^1^	7%	Class I/IIb	HDAC1: 1.6 nM HDAC2: 14 nM HDAC3: 0.5 nM HDAC6: 12 nM	Brain uptake: Rapid brain uptake and limited washout were observed Specific binding in the brain was confirmed in a blocking study (rat)	[79]
[^18^F]**10**	357.46	3.77 ^1^	0.9%	Class I/IIb	HDAC1: 0.8 nM HDAC2: 6.4 nM HDAC3: 0.5 nM HDAC6: 9.5 nM	Brain uptake: Rapid brain uptake and limited washout were observed Specific binding in the brain was confirmed in a blocking study (rat)	[79]
[^18^F]**11**	371.49	4.17 ^1^	0.3%	Class I/IIb	HDAC1: 0.8 nM HDAC2: 7.0 nM HDAC3: 0.8 nM HDAC6: 12 nM	Brain uptake: Rapid brain uptake and limited washout were observed Specific binding in the brain was confirmed in a blocking study (rat)	[79]
[^18^F]**12**	345.45	–	8.1% ^2^	HDAC6	HDAC6: 60 nM Others: ≥1 μM	Brain uptake: Rapid brain uptake and limited washout were observed Specific binding in the brain was confirmed in a blocking study (rat)	[80]

^1^ Clog *P*. ^2^ Non-decay corrected. MW: molecular weight; RCY: radiochemical yield (decay corrected).

**Table 3:** Physiochemical properties of radiolabeled carboxylic acid- and hydroxamic acid-based HDAC ligands.

Compound	MW	Log *D*	RCY	Target	IC_50_ for HDACs	In Vivo Properties in Rodents	Reference
[^11^C]**13**	87.11	1.02	31–50%	Class I	HDAC1: 16 μM HDAC2: 12 μM HDAC3: 9 μM HDAC8: 15 μM	–	[86,87]
[^11^C]**14**	149.18	−0.20	40–55%	Class I	HDAC1: 64 μM HDAC2: 65 μM HDAC3: 260 μM HDAC8: 93 μM	–	[86,87]
[^11^C]**15**	143.21	0.26	38–50%	Class I	HDAC1: 39 μM HDAC2: 62 μM HDAC3: 161 μM HDAC8: 1103 μM	–	[86,87]
[^11^C]**16**	331.37	2.03 ^1^	9% ^3^	HDAC1–3/6	HDAC1: 5 nM HDAC2: 32 nM HDAC3: 3.4 nM HDAC6: 5 nM	Brain uptake: Around 0.1% ID/cc immediately after i.v. injection (rat)	[89]
[^11^C]**17**	348.43	2.66 ^1^	3% ^3^	HDAC1–3/6	HDAC1: 0.2 nM HDAC2: 1.2 nM HDAC3: 0.4 nM HDAC6: 1.6 nM	Brain uptake: Around 0.15% ID/cc immediately after i.v. injection (rat)	[89]
[^11^C]**18**	295.33	2.61 ^1^	5% ^3^	HDAC8	HDAC8: 18 nM	Brain uptake: Around 0.2% ID/cc immediately after i.v. injection (rat)	[89]
[^11^C]**19**	334.41	1.33 ^2^	8%	HDAC6	HDAC1: 5.2 μM HDAC6: 1.4 nM	Brain uptake: Radioactivity peaked in forebrain at 0.44 SUV and in cerebellum at 0.48 SUV (rat)	[93,94]
[^11^C]**20**	334.41	1.33 ^2^	16%	HDAC6	HDAC6: 4 nM Others: ≥1.3 μM	–	[82,95]
[^64^Cu]**21**	1009.02	–	≥98%	Class I/IIb/III	Class I, IIb, III: 94 nM	Brain uptake: Biodistribution study was performed but brain uptake was not assessed (tumor-bearing mouse)	[96]

^1^ Log *p*. ^2^ clog *D*. ^3^ Non-decay corrected. MW: molecular weight; RCY: radiochemical yield (decay corrected).

**Table 4:** Physiochemical properties of radiolabeled benzamide-based HDAC ligands.

Compound	MW	Log *D*	RCY	Target	IC_50_ for HDACs	In Vivo Properties in Rodents	Reference
[^11^C]**22**	375.42	1.8	25%	Class I	HDAC1: 60 nM HDAC2: 153 nM	Brain uptake: <0.10% ID/cm^3^ after 3 min Metabolism: 80% of radioactivity in the brain was unchanged [^11^C]**22** (rat)	[101,105]
[^11^C]**23**	268.30	1.0	10–15%	Class I	HDAC1: 45 nM HDAC2: 31 nM HDAC3: 20 nM	–	[105]
[^11^C]**24**	344.45	2.1	40%	Class I	HDAC1: 10 nM HDAC2: 20 nM	–	[105]

MW: molecular weight; RCY: radiochemical yield (decay corrected).

**Table 5:** Brain PET imaging studies of HDAC radioligands in NHPs.

Compound	Target	Findings in PET Studies in NHPs	Reference
[^18^F]**1**	Class IIa	Radioactivity accumulation was brain region specific in rhesus macaques (~0.03% ID/g) By 30 min p.i., almost all [^18^F]**1** was metabolized to [^18^F]FACE Radioactivity in the brain was decreased by pre-treatment with SAHA in a dose-dependent manner	[26,46]
[^11^C]**6** [^11^C]**7**	–	Brain uptake of both ligands was very low in baboons (~0.004% ID/cc) The unchanged fraction of both ligands in baboon plasma was less than 20% at 30 min p.i.	[60]
[^11^C]**8**	Class I/IIb	Regional *V*_T_ (90-min scan) in the baboon brain ranged from 29.9 to 54.4 mL/cm^3^ Parent fraction in plasma decreased gradually (50% at 30 min p.i. and 40% at 60 min p.i.) The mean *V*_T_ in the brain decreased by 82.3 ± 5.5% with a 1-mg/kg blocking dose	[28,68,78]
[^18^F]**9** [^18^F]**10** [^18^F]**11**	Class I/IIb	Whole brain SUV_30–60 min_ of [^18^F]**9**, [^18^F]**10**, and [^18^F]**11** in baboons were 0.57, 1.2, and 1.8, respectively (2.3 for [^11^C]**8**) [^18^F]**11** showed the highest correlation in regional brain distribution with [^11^C]**8**	[79]
[^18^F]**12**	HDAC6	Excellent brain uptake (SUV ≈ 3 around 30 min p.i.) was observed in baboons Nonspecific binding in the brain determined with 1 mg/kg unlabeled 12 was low (<1 SUV)	[80]
[^11^C]**13** [^11^C]**14** [^11^C]**15**	Class I	In the baboon brain, uptake of the three ligands was low (~0.006% ID/cc)	[86]
[^11^C]**16** [^11^C]**17**	HDAC1–3/6	Brain uptake in baboons was very low over the 80-min scan time	[89]
[^11^C]**18**	HDAC8	Brain uptake in baboons was very low over the 80-min scan time	[89]
[^11^C]**22**	Class I	Brain uptake in baboons was very low (<0.001% ID/cc) over the 90-min scan time Approximately 60% of the plasma radioactivity was unchanged ligand at 40 min p.i.	[101]
[^11^C]**23** [^11^C]**24**	Class I	Total *V*_T_ values of [^11^C]**23** and [^11^C]**24** in the baboon brain were 0.41 and 12 mL/cm^3^, respectively The degree of *V*_T_ reduction of [^11^C]**24** by unlabeled 24 or SAHA (1 mg/kg) ranged from 8–24% in various brain regions	[105]
